# Properdin and Factor H: Opposing Players on the Alternative Complement Pathway “See-Saw”

**DOI:** 10.3389/fimmu.2013.00093

**Published:** 2013-04-23

**Authors:** Lubna Kouser, Munirah Abdul-Aziz, Annapurna Nayak, Cordula M. Stover, Robert B. Sim, Uday Kishore

**Affiliations:** ^1^Centre for Infection, Immunity and Disease Mechanisms, Biosciences, School of Health Sciences and Social Care, Brunel UniversityLondon, UK; ^2^Department of Pharmacology, University of OxfordOxford, UK; ^3^Centre for Biotechnology and Bioinformatics, School of Life Sciences, Jawaharlal Nehru Institute for Advanced StudiesSecunderabad, Andhra Pradesh, India; ^4^Department of Infection, Immunity and Inflammation, University of LeicesterLeicester, UK; ^5^Faculty of Science, Engineering and ComputingKingston upon Thames, Surrey, UK

**Keywords:** properdin, factor H, biosynthesis, complement, extrahepatic

## Abstract

Properdin and factor H are two key regulatory proteins having opposite functions in the alternative complement pathway. Properdin up-regulates the alternative pathway by stabilizing the C3bBb complex, whereas factor H downregulates the pathway by promoting proteolytic degradation of C3b. While factor H is mainly produced in the liver, there are several extrahepatic sources. In addition to the liver, factor H is also synthesized in fetal tubuli, keratinocytes, skin fibroblasts, ocular tissue, adipose tissue, brain, lungs, heart, spleen, pancreas, kidney, muscle, and placenta. Neutrophils are the major source of properdin, and it is also produced by monocytes, T cells and bone marrow progenitor cell line. Properdin is released by neutrophils from intracellular stores following stimulation by *N*-formyl-methionine-leucine-phenylalanine (fMLP) and tumor necrosis factor alpha (TNF-α). The HEP G2 cells derived from human liver has been found to produce functional properdin. Endothelial cells also produce properdin when induced by shear stress, thus is a physiological source for plasma properdin. The diverse range of extrahepatic sites for synthesis of these two complement regulators suggests the importance and need for local availability of the proteins. Here, we discuss the significance of the local synthesis of properdin and factor H. This assumes greater importance in view of recently identified unexpected and novel roles of properdin and factor H that are potentially independent of their involvement in complement regulation.

## Introduction

The complement system consists of various plasma proteins that circulate in the serum, such as zymogens, which upon stimulation interact with each other to opsonize pathogens and promote their removal by phagocytosis. Complement activation takes place via three pathways – classical, lectin, and alternative pathways. The classical pathway is activated when its recognition component C1 binds to a target, via C1q. C1q recognizes targets mainly by surface charged clusters, and binds directly to many bacteria, apoptotic cells, or surfaces which have IgG or IgM attached (Kishore and Reid, 1999; Arlaud and Thielens, 2010). The complement protein complex C1 is made up of three proteins, C1q, C1r, and C1s. Upon binding of the C1q protein to a target, C1r and C1s, which are protease zymogens, become activated and C1s cleaves the C4 and C2 complement components. C4 is cleaved into C4a and C4b. C4a is an anaphylatoxin that causes inflammation and C4b attaches to the target surface. C1s cleaves C2 into C2a and C2b, resulting in the formation of C3 convertase C4bC2a, which generates C3b by cleaving C3. Subsequently, C5 convertase (C4b2a3b) is formed resulting in the formation of the membrane attack complex (MAC) and lysis (Kishore and Reid, 1999; Fujita, 2002; Carroll and Sim, 2011).

The activation of the complement system via the lectin pathway occurs as a result of carbohydrate and other moieties on target surfaces being bound by mannose binding lectin (MBL), ficolins, or collectin-11 (recognition subcomponents of the lectin pathway) (Sim and Laich, 2000; Fujita, 2002; Cestari Idos et al., 2009). These recognition proteins are associated with the protease zymogens MBL-associated-serine-protease (MASP)-1, MASP-2, MASP-3, which are orthologs of C1r and C1s. MASP-2, like C1s, functions to cleave C4 into C4a and C4b and C2 into C2a and C2b. Similar to the classical pathway, a C3 convertase complex, C4b2a is formed leading to the generation of C5 convertase (C4b2a3b), which also leads to the formation of the MAC (Fujita, 2002; Carroll and Sim, 2011).

The activation of alternative pathway takes place on various cellular surfaces such as yeasts, bacteria, and parasites, and is also stimulated by antibody-antigen complexes consisting of IgG or IgA (Carroll and Sim, 2011). Recently, mice studies suggested that MASP-3 is involved in the activation of the alternative pathway. The study demonstrated that the activation of the alternative pathway was restored by the addition of recombinant MASP-3, in sera from MASP or MASP/C4 gene deficient mice. This signifies that MASP-3 may be an important protease required for alternative complement pathway (Iwaki et al., 2011).

Properdin and factor H are key regulatory proteins of the alternative pathway (Kemper and Hourcade, 2008). The initiation of the alternative pathway is triggered by a conformational change in C3 cleaved at a single site by the serine protease C3 convertase (Carroll and Sim, 2011). This leads to the generation of C3a, an anaphylatoxin, and C3b. Nascent C3b binds covalently to surfaces (e.g., of bacterial or fungal cells) through a reactive thioester moiety. Factor B (a protease zymogen) can then associate with C3b in the presence of Mg^2+^. Once the C3bB complex has formed factor D, a serine protease, cleaves factor B into Ba and Bb fragments. The Bb fragment remains bound to C3b generating the C3 convertase C3bBb of the alternative pathway. C3bBb is not very stable under physiological conditions and has half life of about 90 s. This complex is stabilized by binding of properdin (to form C3bBbP), which increases its half life by 5–10-fold (Pillemer et al., 1954; Le et al., 2007). In this amplification loop, further C3b molecules are generated, which are deposited adjacent to the convertase leading to opsonization and the formation of C5 convertase, cleaving C5 into C5a and C5b. This leads to the lytic pathway and cell lysis. On the other hand, the activation cascade is downregulated on host cells due to control by membrane-bound regulatory proteins, factor H, and factor I. Human factor H inhibits the C3 convertase formation by binding to C3b. Together with factor I, factor H cleaves C3b to iC3b. In addition it increases the convertase decay activity, thus inhibiting the activation of complement alternative pathway (Sim et al., 1993).

## Properdin Structure and Function

**Figure 1:**
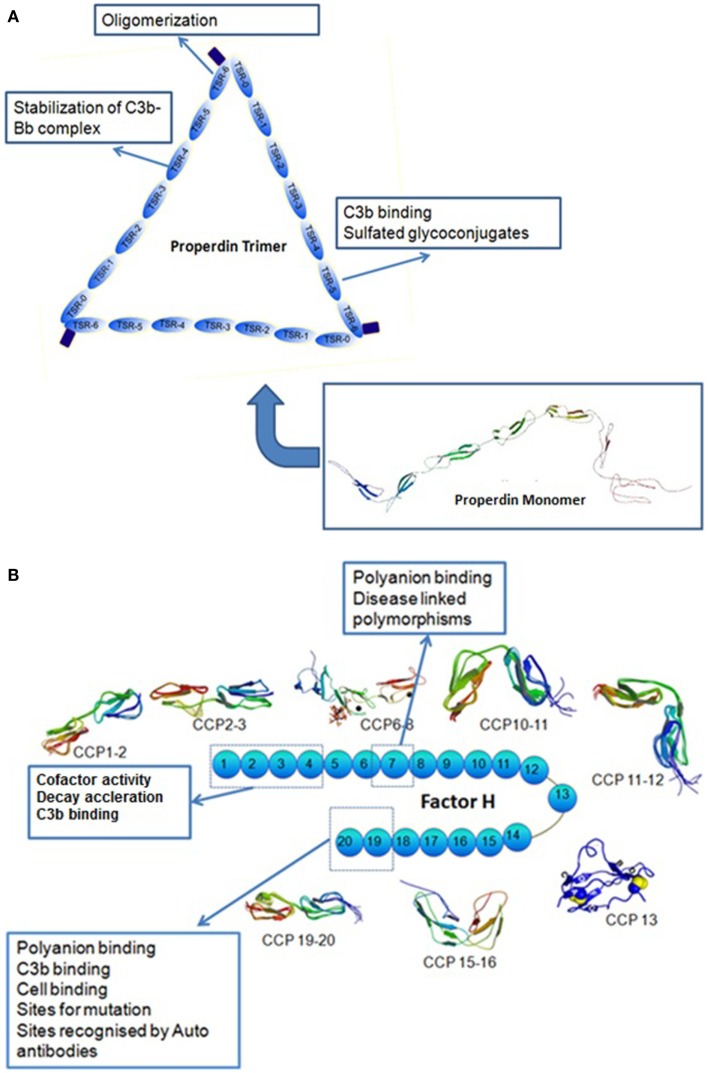
**(A)** Modular organization of thrombospondin repeats in properdin. Properdin can be found in serum in different forms: monomers, dimers, and trimers, tetramers. A molecule of properdin trimer is shown in this figure along with the schematic representation of the MONOMER with its TSRs. TSR0 is the N-terminal TSR. The brown rectangle on the end of the TSR6 represents an N-glycosylation site. TSR4 has been shown to be involved in the stabilization of the C3 convertase. TSR6 is involved in the oligomerization of properdin monomers and TSR5 binds to C3b (figure adapted from Sun et al., 2004 and data from Higgins et al., 1995). **(B)** Illustration of a factor H molecule. Factor H has 20 CCP modules that demonstrate different binding properties. The molecular structures for CCP 1–2, 2–3, 6–8, 10–11, 11–12, 13, 15–16, 19–20 have been established experimentally. Human factor H decays Bb from C3b and is a cofactor for degradation of C3b to iC3b by serine protease factor I. It attaches to surface through its heparin/anionic binding sites in its carboxyl-terminus of short complement repeats (CCP 16–20) while its decay-accelerating activity and cofactor activity sites are at the amino-terminus (CCP 1–4) (Based on Atkinson et al., 2007).

Properdin is a soluble glycoprotein found in plasma at a concentration of about 25 μg/ml composed of several identical subunits of 53 kDa binding to each other in a head to tail manner (Discipio, 1982; Nolan et al., 1992) to form cyclic polymers (dimers, trimers, and tetramers) distributed in plasma in a ratio of 26:54:20 (Smith et al., 1984). The monomer is a flexible rod-like structure with a length of 26 nm and a diameter of 2.5 nm The human properdin monomer is 442 amino acids long and is composed of six (or seven, see below) non-identical tandem repeats of about 60 amino acids called thrombospondin type I (TSR) repeats (Goundis and Reid, 1988; Higgins et al., 1995) TSRs are also found in cell adhesion molecules in human blood platelets called thrombospondins. Initially each monomer of properdin was thought to have five and a half TSR domains, each having 58–67 residues with an addition of N-terminal and C-terminal domains (Sun et al., 2004). However, further sequence alignments indicated that there is a sixth TSR domain which is larger than the rest due to a sequence insertion. More recent work proposes that properdin has seven thrombospondin type I repeats instead of the six, as the N-terminal domain (TSR-0, a truncated domain) has the same six conserved Cys residues established in TSR1–TSR6 (Sun et al., 2004) (Figure 1A).

The human properdin gene has 10 exons with a length of ∼ 6 kb (Nolan et al., 1992). The first exon is untranslated, the second exon consists of the translation start site as well as a sequence which encodes 24 amino acids of signal peptide and the N-terminal region is encoded by exon 3. Exon 4–8 encode each of the TSRs 1–5, exon 9 encodes the first 38 amino acids of TSR6 with the remaining part of TSR6 encoded by exon 10 and the C-terminal region is also encoded by exon 10 (Nolan et al., 1992; Higgins et al., 1995).

Domain deletion studies (Higgins et al., 1995) have shown that properdin lacking TSR4 forms dimers and binds to C3b, but not to C3bBb. Properdin without TSR5 does not bind to C3b and so for both deletions the C3bBb complex is not stabilized (Higgins et al., 1995). Properdin lacking TSR3 is still able to form oligomers (dimers, trimers, tetramers) and also monomers. This deletion does not prevent properdin from binding C3b and the C3bBb complex. TSR4 and TSR5, therefore, appear to play an important role in the binding of C3b, sulfatides, and in the stabilization of C3 convertase of the complement alternative pathway. TSR5 is involved in the binding of sulfated glycoconjugates but the binding region for C3bBb is different (Perdikoulis et al., 2001). Deletion of TSR6 and the short C-terminal charged tail prevents the formation of oligomers, and the monomers cannot stabilize C3bBb, bind C3b, or sulfatide (Higgins et al., 1995).

Recent studies have shown that properdin (as well as factor H) can recognize glycosaminoglycan (GAG), with properdin binding to more GAG epitopes than factor H, which requires sulfated motifs on GAGs for interaction. These interactions by properdin and factor H reported for renal tubular heparin sulfates involve overlapping epitopes and can be modulated by anticoagulant heparinoids (Zaferani et al., 2012). It has been demonstrated that apoptotic T cell recognition by properdin is via GAG-binding site (Kemper et al., 2008). Heparin interacts with properdin (and factor H) but the binding kinetics and dissociation constants (*K*_d_) are dissimilar (Yu et al., 2005). Properdin has also been shown to interact with sulfated glycoconjugates dextran sulfate and fucoidan (Holt et al., 1990).

Though studies have shown interaction of properdin with mammalian cells via GAGs, there are no GAGs present on *Neisseria* or enteric bacteria, demonstrating that properdin also interacts with other biochemical targets on microbial surfaces. Studies have demonstrated the binding of properdin to *Salmonella typhosa* lipopolysaccharide (LPS) and *Neisseria meningitidis* LOS induce activation of the complement alternative pathway (Kimura et al., 2008). Properdin has been reported to bind directly to microbial surfaces, recruiting fluid phase C3b and so initiating the assembly of the alternative pathway C3 convertase. Spitzer et al. (2007) reported that properdin binds to wild-type *Neisseria gonorrhoeae*, allowing the C3 convertase (C3bBbP) complex to form, which leads to opsonization of bacteria. This suggests that properdin can directly mediate the assembly of alternative pathway on microbial surfaces (Spitzer et al., 2007) (Table 1). However it is possible that unfractionated properdin undergoing freeze thawing consists of high order oligomers of properdin, which may bind surfaces that native properdin would not (Farries et al., 1987). Agarwal et al. (2010), however, found that properdin does not bind directly to *N. meningitidis* or *N. gonorrhoeae* but enhances the deposition of C3 on the bacterial surface by stabilizing the alternative pathway C3 convertase. Another report has shown that native properdin (dimer, trimer, tetramer) binds to *Chlamydia pneumonia*. The binding of properdin to *C. pneumonia* increased C3b deposition and induced complement activation (Cortes et al., 2011).

**Table 1:** **Known functions of properdin**.

Functions of properdin	Reference
Properdin assembles C3bBb on a surface by promoting the association of C3b and factor B, and also binds C3 convertase	Pillemer et al. (1954)
Binding of properdin to C3bBb complex allows it to be stabilized as it increases its half life of about 5–10-folds	Hourcade (2006)
Direct binding of properdin to microbial organisms such as zymosan on yeast cell walls and *N. gonorrhoeae* results in *in situ* assembly and function C3 convertases resulting in opsonization and elimination of pathogen	Pillemer et al. (1954)
Properdin binds to zymosan and this complex binds C3b.	Spitzer et al. (2007)
Properdin also binds to erythrocytes, which leads to the generation of Er-C3bBbP complexes	Cortes et al. (2011)
Properdin binds independently of C3b to late apoptotic cells and necrotic cells. This direct binding is crucial for the local amplification of the complement alternative pathway activation.	Xu et al. (2008)
Properdin enhances apoptotic T cell uptake by macrophages and dendritic cells.	Kemper et al. (2008)

## Factor H Structure and Function

Human factor H is an extended glycoprotein (Sim and DiScipio, 1982) of 155 kDa. It is thought to be mainly monomeric, but may have some dimers in the circulating population (Perkins et al., 2012). It consists of 20 complement control protein (CCP) domains, each consisting of 60 amino acids (Ripoche et al., 1988a). Human factor H contains three different binding sites for C3b or C3d throughout its length with CCP 1–4 being the major site as well as CCP 12–14 and CCP 19–20 (Alsenz et al., 1985; Gordon et al., 1995; Jokiranta et al., 2000) (Figure 1B). The protein can be found in the plasma at a concentration of ∼200–700 μg/ml (Kishore and Sim, 2012). Its main function is to distinguish between endogenous and exogenous particles or surfaces and to limit the activation of C3. Human factor H appears to bind multiple sites in C3 and has been shown to have a higher apparent binding avidity for C3b bound to non-activators of the alternative pathway compared to C3b bound to activators. This is thought to occur because factor H binds to negative charge clusters such as sialic acids or GAGs which cover mammalian cells, flagging them as non-activators. Factor H can, therefore, bind to both the polyanionic structures as well as C3b, resulting in a higher apparent avidity for C3b bound to a non-activator surface (Meri and Pangburn, 1990).

A number of proteins which are closely related in structure to factor H, also circulate in plasma. These are factor H-like protein-1 (FHL-1) and factor H-related proteins 1–5 (FHR1-5) (Zipfel et al., 2002). FHL-1 is also known as reconectin and consists only of seven CCP domains followed by the amino acid sequence SFTL. It arises as result of alternative splicing of the factor H gene. Its CCP 1–7 are identical to those of factor H (Ripoche et al., 1988a). FHRs 1–5, each of which is encoded by a separate gene in the regulation of complement activation (RCA) cluster, have not been functionally annotated fully, but FHR-3 and FHR-5 both bind C3b and FHR-3 also binds heparin (Estaller et al., 1991; Hellwage et al., 1999; McRae et al., 2001; Zipfel et al., 2002).

**Figure 2:**
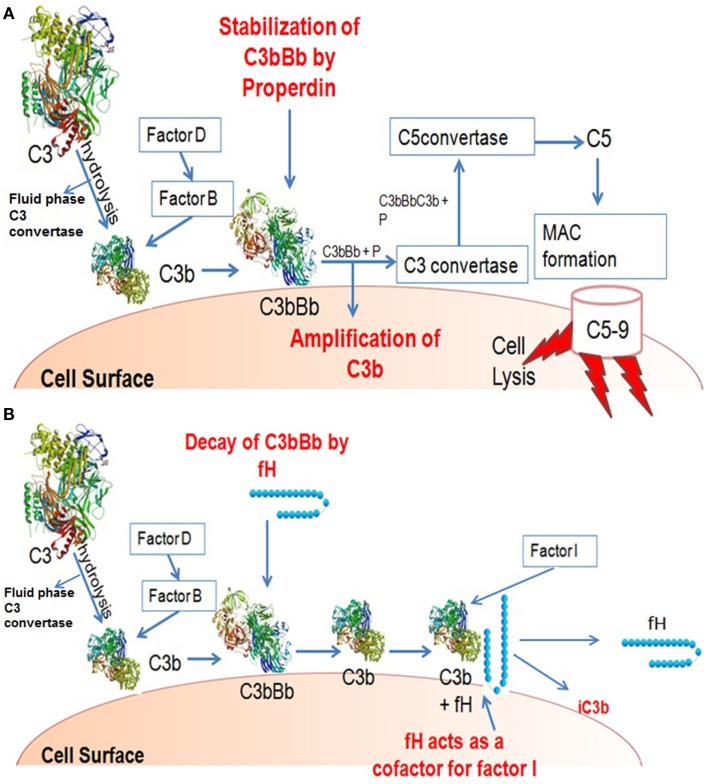
**Illustration of activities of factor H and properdin**. **(A)** Properdin can be found in serum in different forms: monomers, dimers, and trimers. A Properdin stabilizes C3 convertase that cleaves more C3 to C3b thus amplifying the process. **(B)** C3b bound on a surface can bind factor B. C3b-factorB is cleaved by factor D to form C3bBb. Factor H can displace Bb from its binding to C3b, and form a C3b-factor H complex. C3b-factor H is cleaved by factor I to form iC3b; with the release of factor H, iC3b does not bind factor B so cannot form a convertase like C3bBb.

Human factor H functions as a downregulator of the alternative pathway activation. It obstructs the formation of the alternative pathway C3 convertase and enhances the decay of the convertase (decay acceleration activity) by dissociating Bb from the C3 convertase complex and C5 convertase complex, thus inhibiting the positive feedback loop (i.e., the amplified turnover of C3). The formation of alternative pathway C3 convertase can also be inhibited by the binding of factor H to C3b, hence inhibiting the interaction of C3b and factor B (Sim et al., 1993). It acts as a cofactor for factor I for the cleavage of C3b to iC3b (cofactor activity). Mutations resulting to factor H functional deficiency can cause uncontrolled alternative pathway activation, as is the case in dense deposit disease (DDD) patients (Zhang et al., 2012). Whereas human factor H functions to downregulate alternative pathway, properdin up-regulates by stabilizing the C3 convertase, thus generating C3b molecules and leading to opsonization and the formation of lytic pathway (Pangburn and Muller-Eberhard, 1984) (Figures 2A,B). FHR4 demonstrates qualitatively similar complement regulatory activity to factor H, in both decay-accelerating and cofactor activities (Hellwage et al., 1997).

Recently, factor H has been shown to directly compete with C1q in binding to anionic phospholipids (e.g., cardiolipin) (Tan et al., 2010), lipid A and *Escherichia coli* (Tan et al., 2011) and beta amyloid peptide (Nayak et al., 2010; Nayak et al., unpublished) and acts as a direct downregulator of the classical pathway (Table 3). This ability of factor H to dampen classical pathway activation is distinct from its role as an alternative pathway downregulator. Thus, by directly competing with C1q to bind to self and non-self ligands (Kishore et al., 2004), factor H is likely to be involved in regulating C1q-mediated complement activation inflammatory processes in autoimmunity and infection. It is interesting that factor H binds to apoptotic cells, another C1q target, but has no effect on the uptake of apoptotic cells by phagocytes. However, C1q-mediated enhancement of uptake/adhesion of the apoptotic cells by monocytes is considerably reduced by factor H (Kang et al., 2012). Thus, factor H may be important in controlling the inflammation, which might arise from C1q deposition on apoptotic cells.

A number of pathogens have acquired the ability to bind factor H, via charge interactions (Lambris et al., 2008). *N. meningitidis*, for example, has been shown to bind factor H probably to allow the organism to evade the complement system. Two *N. meningitidis* ligands, called factor H binding protein (FHbp) (Schneider et al., 2009) and Neisserial surface protein A (NspA) (Madico et al., 2006; Lewis et al., 2010, 2012) have been identified.

## Extrahepatic Biosynthesis of Properdin

The majority of the complement pathway proteins are synthesized in the liver. Extrahepatic biosynthesis of complement proteins occurs in a wide variety of cells, e.g., in monocytes, fibroblasts, neuronal cells, adipose tissue, and endothelial cells (EC) (Friese et al., 1999). Properdin however, along with a few other complement proteins, i.e., C1q, serine protease factor D and C7, have different primary sources of synthesis. Neutrophils are the major source of properdin. Monocytes, bone marrow progenitor cell lines and T cells also produce properdin (Wirthmueller et al., 1997).

Human monocytes have been shown to secrete alternative pathway complement proteins including properdin (Whaley, 1980). The biosynthesis of properdin in response to LPS, phorbol 12-myristate 13-acetate (PMA) and various cytokines has been detected in the Mono-Mac-6 cell line, which has morphologically and functionally similar to mature peripheral blood monocytes (Schwaeble et al., 1994). TNF-α and IL-1ß enhance mRNA abundance and secretion of properdin, whereas IFN-γ downregulates it (Schwaeble et al., 1994).

## Properdin Biosynthesis by Different Cell Types

Maves and Weiler (1994) reported that functional properdin is a product of human liver-derived HEP G2 cells. Although the major extrahepatic source of properdin is neutrophils, the source of properdin in circulation is not known. Human liver-derived HEP G2 cells can produce functional properdin (Maves and Weiler, 1994). Properdin localized in the granules of human neutrophils are secreted when stimulated with TNF, TNF/fMLP, PMA, C5a, or IL-8. This suggests that neutrophils can promote complement activation upon stimulation with cytokines or coagulation-derived factor, thus stabilizing and amplifying the alternative pathway via release of properdin. This allows the activation of the alternative pathway leading to defense against invading microorganisms (Wirthmueller et al., 1997; Camous et al., 2011). Properdin concentration in plasma is low in comparison to other alternative pathway components [C3: 1000–1500 μg/ml (Kohler and Muller-Eberhard, 1967); and factor B: 74–286 μg/ml] (Oglesby et al., 1988). Thus, properdin secretion by neutrophils is an important source for local alternative pathway activity.

The alternative pathway is activated by locally produced properdin by neutrophils, which in an amplification loop, releases C5, which further augments neutrophil pro-inflammatory responses (Camous et al., 2011). The release of anaphylatoxin C5a mediates chemotaxis and degranulation of neutrophils secreting properdin, implying that neutrophils support the complement activation system facilitating elimination of various microorganisms (Schwaeble and Reid, 1999). However, uncontrolled activation of the alternative pathway via neutrophil secreted properdin could potentially promote inflammatory responses, which may be harmful for the host (Sylvestre and Ravetch, 1994) leading to diseases such as vasculitis and rheumatoid arthritis (Schwaeble and Reid, 1999). Properdin, C2, and factor D deposits in the synovium are also involved in the inflammatory process (Dimitrova et al., 2010). Mice studies have shown that properdin in the systemic circulation promotes alternative pathway –mediated injury in arthritis, making properdin a possible therapeutic target. Release of properdin locally by leukocytes at the site of inflammation would activate the alternative pathway and aggravate tissue injury in the joint (Kimura et al., 2010).

Properdin secreted by activated neutrophils binds to apoptotic T cells without activating complement since the concomitant C3b deposition is not detected on T cells or neutrophils. The granules of neutrophils secrete properdin, which then binds to apoptotic cells (Kemper et al., 2008) (Table 2). In addition, both CD4^+^ and CD8^+^ T cells have the potential to produce properdin (Schwaeble et al., 1993). Properdin binds to apoptotic T cells and enhances phagocytosis by macrophages, suggesting that properdin functions in the recognition via sulfated GAGs and removal of apoptotic cells (Kemper and Hourcade, 2008; Xu et al., 2008; Ferreira et al., 2010). Dendritic cells, like macrophages, can also be a source of complement proteins, i.e., properdin, factor H, factor I, C3, C5, C9, factor D, and C1q (Reis et al., 2007). Dendritic cells found in most tissues are important in circumstances where complement proteins may be at very low concentration, as dendritic cells play a role in capturing, processing, and presenting antigens to T lymphocytes.

**Table 2:** **Local (extra hepatic) biosynthesis of properdin**.

Sites/source of biosynthesis of properdin	Comments/possible implications	Reference
Neutrophils	Properdin is localized in the granules of neutrophils and released by TNF, TNF/fMLP, PMA, C5a, or IL-8	Camous et al. (2011)
T cells	Promotes phagocytosis of apoptotic T cells, suggesting that properdin functions in the recognition and removal of apoptotic cells. Drives the uptake of apoptotic cells by macrophages and dendritic cells.	Kemper and Hourcade (2008)
Endothelial cells	Properdin up-regulation is induced by shear stress of 2–3 dyn/cm^2^	Bongrazio et al. (2003)
Peripheral blood monocytes	Synthesize properdin, *in vivo* plays a crucial role in alternative pathway mediated injury	Whaley (1980)

**Table 3:** **Various functions assigned to factor H**.

Functions of factor H	Reference
Downregulates the alternative pathway.	Whaley and Ruddy (1976)
Acts as a cofactor for factor I in the process of conversion of C3b to iC3b	
Inhibits the formation of C3 convertases of the alternative pathway by binding to C3b	Conrad et al. (1978)
Prevents the interaction of C3b and factor B	
Decay acceleration activity: inhibits alternative pathway by dissociating C3 convertases C3bBb and C3bBbC3b	Weiler et al. (1976)
Downregulates classical pathway by competing with C1q for binding to activators.	Kishore and Sim (2012)
Have roles as adhesion ligands in host-pathogen interactions.	Losse et al. (2010)
Neutrophils exhibit a cellular receptor CR3 for factor H, CFHL1, and for ComoloR1 that enhances attachment of neutrophils to *C. albicans*	Losse et al. (2010)

Endothelial cells, at the inner surface of the blood vessel wall, express a range of complement proteins. Properdin transcripts are induced in ECs in response to shear stress, following which properdin is synthesized and released in extracellular compartments. Shear stress of 2–3 dyn/cm^2^ can induce properdin up-regulation (Bongrazio et al., 2003). Furthermore, changes in the shear stress could potentially result in local changes in the concentration of properdin, thus regulating the alternative pathway (Bongrazio et al., 2003).

Properdin has also been implicated in energy metabolism and lipid metabolism (Gauvreau et al., 2012). In the properdin gene deficient mice, properdin deficiency leads to fat storage, decrease in energy output and the post-prandial triglyceride clearance delay, compared to the wild-type mice. Thus, properdin could regulate fatty acid uptake into adipose tissue suggesting that complement may have a dual role in adipose tissue (Gauvreau et al., 2012).

The alternative pathway and complement proteins may be involved in the cartilage transformation. The distribution of properdin on tibia appears in the resting area but not in all cells, and is also found in the hypertrophic area localized at the cell periphery. C3, factor B and properdin are observed in the resting zone of cartilage, indicating synthesis of these proteins by cartilage cells. Furthermore the presence of these proteins in resting cartilage may indicate that the alternative pathway could possibly play a role in the development of this tissue (Andrades et al., 1996).

## Extrahepatic Biosynthesis of Factor H

The liver is the primary site of factor H production. The liver expresses two different human factor H mRNA species; the 4.3 kb (FH) and the 1.8 kb (FHL-1) (Estaller et al., 1990). The 4.3 kb mRNA is more abundant than the 1.8 kb. The factor H secretion by Kupffer cells and hepatocytes is up-regulated by IFN-γ in rats (Schlaf et al., 2002). The extrahepatic synthesis of factor H in humans is also up-regulated by IFN-γ (Gasque et al., 1995; Friese et al., 1999, 2003; Schlaf et al., 2001). Low levels of extrahepatic factor H expression have been reported in the brain, eyes, lungs, heart, spleen, kidneys, pancreas, and placenta as well as neurons, and glial cells (Nawajes et al., 2006). Complement proteins are known to be expressed in neurons, microglia, astrocytes, and oligodendrocytes (McGeer and McGeer, 2004). Neurons, which are particularly susceptible to complement-mediated damage, may be protected by factor H synthesis against the classical and the alternative pathway mediated cell damage.

Several components of the alternative pathway are also synthesized in the adipose tissue (Moreno-Navarrete et al., 2010). Adipocytes express factor H and various other proteins, such as properdin, C3, factor B, C1q, and C3a-receptor (C3aR). Factor H gene expression has also been detected in human adipose tissue. Interestingly, circulating factor H corresponded positively with body mass index (BMI), waist circumference, triglycerides, and inflammatory parameters, and negatively with insulin sensitivity and high density lipoprotein (HDL) cholesterol (Moreno-Navarrete et al., 2010). The association between factor H gene expression and insulin resistance was only found in the omental fat. Mature adipocytes also showed significant gene expressions of both factor H and factor B. This increase in circulating factor H and factor B concentration in subjects with abnormal glucose tolerance could reflect the elevated stromovascular cells (SVC)-induced alternative pathway activation in omental adipose tissue linked to insulin resistance and metabolic disturbances (Moreno-Navarrete et al., 2010).

## Local Synthesis of Factor H by Different Cell Types

Complement synthesis and its regulation have also been examined in cell lines derived from hepatomas and myelomonocytic tumors (Perlmutter and Colten, 1986). Additionally, several tumor cells have been reported to express and release increased amounts of factor H, possibly to reduce complement activation in their microenvironment and provide protection from complement attack (Wurzner et al., 1995; Fedarko et al., 2000; Junnikkala et al., 2000). Factor H expression is enhanced by IFN-γ in monocytes, human umbilical vein endothelial cells (HUVEC) and as much as sixfold in synovial fibroblasts but not by TNF-α (Schwaeble et al., 1987). The presence of higher levels of factor H induced by IFN-γ is likely to confer resistance against complement-mediated lysis of these cells (Ripoche et al., 1988b; Friese et al., 1999). Likewise, ECs as well as epithelial cells also secrete factor H, which is augmented by IFN-γ (Ripoche et al., 1988b; Brooimans et al., 1989, 1990). Mesenchymal stem cells (MSCs) are another type of cell that constitutively produce factor H at the rate of ∼300 ng/10^6^ cells/day. Factor H produced by MSCs is also increased by IFN-γ as well as TNF-α (Tu et al., 2010).

Factor H is synthesized by keratinocytes with regulation by INF-γ (Table 4). Keratinocytes are also reported to produce C3 and factor B as well as cytokines (Pasch et al., 1999). It has been suggested that keratinocytes would need to produce complement regulatory proteins as a mechanism to prevent epidermal damage from autologous complement activation in response to invading pathogens (Timar et al., 2006).

**Table 4:** **Extrahepatic sources of factor H**.

Sites/source of biosynthesis of factor H	Comments/possible implications	Reference
Neuronal cells	Protects cells from complement-mediated damage which can causes central nervous system (CNS) injury	McGeer and McGeer (2004)
Endothelial cells	Secretes factor H along with epithelial cells. IFN-γ heightens expression	Brooimans et al. (1989)
Mesenchymal stem cells	Produce factor H constitutively to inhibit complement activity. Regulated by IFN-γ and TNF-α	Tu et al. (2010)
Adipose tissue	Linked to insulin resistance, obesity, and metabolic disorders	Moreno-Navarrete et al. (2010)
Keratinocytes	Modulated by IFN-γ, to maintain homeostasis in order to prevent epidermal damage	Timar et al. (2006)

Factor H, unlike properdin, binds neutrophils via CR3 as well as CR4 and has been shown to induce neutrophil adherence and release of reactive oxygen species (ROS) (DiScipio et al., 1998). Additionally, release of endogenous anti-inflammatory factor H protects synovial fibroblasts against inflammatory damage and complement-mediated cell lysis during and at the onset of rheumatoid arthritis (Friese et al., 2003).

## Factor H and Properdin Interaction with Heparin Sulfate in Proteinuria

Both factor H and properdin bind to different epitopes on heparin sulfate of proximal tubular epithelial cells. CCP 7 (Clark et al., 2006), CCP 9 (Ormsby et al., 2006), and CCP 19–20 modules (Blackmore et al., 1996, 1998) of factor H have been identified as binding sites for polyanions such as heparin. The alternative pathway has a significant role in renal diseases. Renal injury may result from mutations of the complement system. In proteinuria the complement-mediated tubular injury is a result of complement factors found in ultrafiltrate which activates the alternative pathway on tubular cells (Zaferani et al., 2012). Studies on rats showed factor H in the tissue of normal rat kidney, localized in glomeruli and interstitium, particularly in the interstitial extracellular matrix (ECM). During proteinuria, factor H was shown to be localized on the luminal side of tubuli. In tubular brush borders of proximal tubuli, factor H is not present whereas factor H was observed to be localized on the brush borders during proteinuria. Properdin, on the other hand, is localized on the apical side of tubular cells during proteinuria where factor H is also co-localized. However, in some tubuli either properdin or factor H were found to be positive (Zaferani et al., 2012).

The alternative pathway is activated by properdin on tubular cells through heparin sulfate interaction, whereas factor H inhibits the alternative pathway on tubular cells also by binding to heparin sulfate. Therefore, alternative pathway activation would proceed when properdin binds only to heparin with low sulfation, thus up-regulating the alternative pathway. On the other hand, factor H binds to more highly sulfated heparin isoforms, thus downregulating the alternative pathway (Zaferani et al., 2012). The filtered properdin is crucial for complement activation on the tubular surface in patients with proteinuric renal disease. Thus, inhibiting properdin binding and subsequent alternative pathway activation on the tubular cells may have therapeutic value in proteinuric renal disease (Gaarkeuken et al., 2008).

## Types of Properdin Deficiencies

All phenotypes of properdin deficient individuals express mRNA properdin synthesis by monocytes (Fredrikson et al., 1998). Three phenotypes for properdin deficiencies have been identified: type I deficiency (complete deficiency) of properdin is the absence of properdin and function in plasma; type II (incomplete deficiency) is characterized by low concentrations of properdin in plasma which is less than 10% of normal; and type III (dysfunction of properdin) is the rare type which produces impaired fragment of properdin (Fijen et al., 1999). These deficiencies have been linked to the susceptibility to meningococcal disease. Properdin being expressed by monocytes were observed in type I deficiency and in type II deficiency with two cases from Swedish and Danish family (Fredrikson et al., 1998). Thus, in properdin type I deficient individuals, monocytes synthesize no intracellular or secreted properdin. This might be due to a normal mRNA transcription and a truncated properdin molecule being synthesized (Fredrikson et al., 1998). Conversely, intracellular properdin and secreted properdin is produced normally in individuals with type II deficiency. In type II deficient individuals, a low level secretion of properdin by monocytes can take place even in the absence of cytokine stimulation (Schwaeble et al., 1994).

Properdin deficiency can also lead to functional changes in neutrophils and CD4^+^ T cells (Dimitrova et al., 2010), which prevent joint alterations and inflammatory processes, suggesting the role of properdin in immune complex-induced arthritis. A systemic immune response has been evident in wild-type and properdin deficient mice. Bone erosion in zymosan induced arthritis model was seen in wild-type as well as properdin deficient mice that was localized in bone marrow, cartilage, and bone matrix. In the wild-type mice, CD4^+^ T cells were found in all infiltration areas of joints. However, in properdin deficient mice, infiltration areas and cartilage were mainly enriched with C5aR positive cells (very weak staining for CD4 positivity) but decreased synovial levels of C5a compared to wild-type mice. The abundant C5aR staining in properdin deficient mice could be localized on synoviocytes in cartilage, suggesting its contribution in bone degradation. In addition, macrophages and mast cells in the infiltration region are likely source of C5aR, promoting joint inflammation via pro-inflammatory cytokines and mediators. It is possible that over a period, properdin deficiency leads to infiltration of a range of effector cells in a hierarchical way leading to proteoglycan loss and bone erosion (Dimitrova et al., 2010).

## Human Factor H-Associated Diseases

Mutations or polymorphisms found in factor H heparin binding sites result in dysfunction of factor H, associated with over activation of complement causing tissue damage (Clark et al., 2013). DDD or membranoproliferative glomerulonephritis type II is a rare renal disease that progresses to end-stage renal failure in about 50% of patients. The disease is associated with uncontrolled alternative pathway activation in plasma that generates C3 activation fragments depositing in the glomeruli (Smith et al., 2007). Similarly, C3 activation is uncontrolled in factor H-deficient mice and the C3 is accumulated at the glomerular basement membrane. DDD has been associated with mutations of factor H (Dragon-Durey et al., 2004; Licht and Fremeaux-Bacchi, 2009). These mutations may lead to a quantitative factor H deficiency or dysfunctional factor H and, thus, an insufficient control on plasma complement activation (Dragon-Durey et al., 2004). Loss of properdin also aggravates renal disease linked to the deficiency of factor H (Ruseva et al., 2012).

Age-Related Macular Degeneration (AMD) is a leading cause of visual impairment in elderly, western populations. In recent years, complement gene mutations and polymorphisms have been found to be associated with AMD, suggesting that the complement system is involved in the pathogenesis of the disease (Charbel Issa et al., 2011). The common factor H polymorphism 402H has been identified as an important genetic risk factor for developing AMD, while common polymorphisms in C3 and factor B also contribute to the disease. In addition, defects of factor H in the RCA at the surfaces of the Bruch’s membrane in the retina may also result in AMD (Hageman et al., 2005; Clark et al., 2010; Heurich et al., 2011). Curiously, properdin has also been screened in patients with AMD and from the 10 exons sequenced in properdin of patients with AMD, only four single nucleotide polymorphisms (SNPs) were identified. However, three SNPs were intermittent and the fourth SNP of exon 10 is frequent, but not linked with AMD (Seitsonen et al., 2010). Properdin has also been found to be expressed in choroidal neovascular membranes of patients with AMD (Wolf-Schnurrbusch et al., 2009).

Additionally, factor H mutations affect ∼30% of Atypical Hemolytic Uremic Syndrome (aHUS) patients. More than 100 different factor H mutations have been described in aHUS patients (Saunders et al., 2006, 2007). Heterozygous mutations have been described in majority of cases, affecting various domains of factor H. However, most of the mutations affect CCP 19–20 module. Functional analyses of several of these mutants showed an impaired interaction with C3b, heparin, and ECs (Sánchez-Corral et al., 2002; Manuelian et al., 2003; Clark et al., 2013). Additionally, anti-factor H IgG autoantibodies, which affect mainly young patients and children, are detected in ∼10% of aHUS patients (Józsi et al., 2008). Factor H and low levels of C3 are synthesized by glomerular mesangial cells. It has been found that IL-1 induces synthesis of C3, but not factor H. Factor H production is stimulated by IFN-γ in human mesangial cells (van den Dobbelsteen et al., 1994). These cells are involved in the structure and function of normal glomerulus (Couser et al., 1985; Lesher et al., 2013).

Low levels of properdin have been detected in renal diseases due to hypercatabolism. In the glomeruli, deposits of properdin have been identified in patients with the acute and chronic lupus nephritis (Ziegler et al., 1975). In glomerular basement membrane, the presence of properdin granular deposits was identified in DDD (Kim et al., 1979). This indicates that the loss of properdin is also involved in the pathogenesis of renal diseases associated with factor H (Lesher et al., 2013).

## Conclusion

Further studies into the synthesis of these two complement regulatory proteins in different tissues and cell types should give more insight into the role of properdin and factor H as a potential biomarker for a range of diseases. For example, factor H has been shown to be elevated in the bronchoalveolar lavage (BAL) and sputum of lung cancer patients (Pio et al., 2010) and has also been described as a marker for bladder cancer (Cheng et al., 2005). Further studies may shed light on the role of properdin in the pathogenesis of AMD as well as its therapeutic involvement. Underpinning the conditions which regulate enhanced/decreased local synthesis of properdin and factor H by various cells could help understand the pathogenesis of a range of diseases. More importantly, both properdin and factor H are vital complement regulators as well as modulators of cellular functions. These proteins regulate cellular adhesion, phagocytosis, and antimicrobial activities. The non-complement related properties of properdin and factor H merit further investigation in order to understand their homeostatic role in the clearance of apoptotic cells, modulation of adaptive immunity, resistance against infections, and cellular interactions with ECM, and tumor cells.

## Conflict of Interest Statement

The authors declare that the research was conducted in the absence of any commercial or financial relationships that could be construed as a potential conflict of interest.

## Abbreviations

AP, alternative pathway; CCP, complement control protein; DCs, dendritic cells; DDD, dense deposit disease; ECs, endothelial cells; FHbp, factor H binding protein; fMLP, *N*-formyl-methionine-leucine-phenylalanine; HUVECs, human umbilical vein endothelial cells; IFN-γ, Interferon-gamma; IL-1α, interleukin-1 alpha; IL-1β, Interleukin-1 beta; IL-2, interleukin 2; IL-8, interleukin 8; LOS, lipooligosaccharide; LPS, lipopolysaccharides; MAC, membrane attack complex; MSCs, mesenchymal stem cells; NspA, Neisserial surface protein A; PBMCs, peripheral blood mononuclear cells; PMA, phorbol 12-myristate 13-acetate; TGF-ß1, transforming growth factor-ß1; TNF-α, tumor necrosis factor alpha.
